# Relationship Between Alzheimer’s Disease and the Immune System: A Meta-Analysis of Differentially Expressed Genes

**DOI:** 10.3389/fnins.2018.01026

**Published:** 2019-01-17

**Authors:** Nan Wang, Ying Zhang, Li Xu, Shuilin Jin

**Affiliations:** ^1^Department of Mathematics, Harbin Institute of Technology, Harbin, China; ^2^Department of Pharmacy, Heilongjiang Province Land Reclamation Headquarters General Hospital, Harbin, China; ^3^College of Computer Science and Technology, Harbin Engineering University, Harbin, China

**Keywords:** Alzheimer’s disease, hippocampus, immune pathways, meta-analysis, pathway analysis

## Abstract

Alzheimer’s disease (AD), a neurodegenerative diseases (neuro-diseases) which is prevalent in the elderly and seriously affects the lives of individuals. Many studies have discussed the relationship between immune system and AD pathogenesis. Here, the meta-analysis of differentially expressed (DE) genes based on microarray data was conducted to study the association between AD and immune system. 9519 target genes of hippocampus in 146 subjects (73 AD cases and 73 controls) from 4 microarray data sets were compiled and DE genes with p < 1.00E - 04 were selected to conduct the pathway-analysis. The results indicated that the DE genes were significantly enriched in the neuro-diseases as well as the immune system pathways.

## Introduction

Alzheimer’s disease (AD), the common shared and complicated neurodegenerative disorder, is characterized as functional impairment, progressive cognitive dysfunction, and memory loss in the elderly (Pedersen, 2010; Hu et al., 2016). It is likely caused by a series of complex interactions of environmental, lifestyle and genetic factors. About 70% of the risk is believed to be genetic (Girard et al., 2018). However, the specific genes that contribute to AD are largely unknown and a great deal of effort has been put into detecting the genetic determinants of AD. There are many methods to investigate the pathogenesis of AD (Cheng et al., 2016a; Yang et al., 2016; Liang et al., 2018) and we used meta-analysis of microarray data to explore the differentially expressed (DE) genes associated with AD (Evangelou and Ioannidis, 2013). Since 2009–2017, large-scale AD studies reported the related impact of genes such as CLU, BIN1, CR1, MS4A6E/MS4A4, PICALM, EPHA1, CD2AP, TREM2, DRB1/ HLA-DRB5, SORL1, SLC24A4-0RING3, PTK2B, MEF2C, DSG2, INPP5D, NME8, FERMT2, CELF1, GAB2, CASS4, and ZCWPW1 (Jiang et al., 2017; Hu et al., 2017a). Recent studies discovered AD was related to genes including WWC1, ABCA7, APOE, CD33 TRIM22, FOXO3, PP4R3A, DAPK1 (Christopher et al., 2017; Cheng et al., 2018b; Chung, 2018; Gusareva et al., 2018; Moreno-Grau et al., 2018). However, some genes like IGF1 and IGFBP3 may not be contacted with AD (Williams et al., 2017; Hu et al., 2017b, 2018).

Amyloid hypothesis and neurofibrillary tangles have been considered as the most important pathogenesis of AD in the past decades (Heppner et al., 2015; Cheng et al., 2016b). The sequences of AD based on amyloid hypothesis are the disposition of the astrogliosis and amyloid-β peptide, then the presence of neurofibrillary tangles which are largely composed of tau fragments and hyperphosphorylated tau protein, and eventually the loss of neuronal and synaptic function. Inflammatory response is also a character of AD and escalates with disease progression (Hardy and Selkoe, 2002). The relationship between immune system and AD has not attracted much attention in until recently. However, new research suggests that the immune system, a host defense system, is related with the pathogenesis of AD (Blasko and Grubeck-Loebenstein, 2003; Cheng et al., 2017). The importance of immune system pathways for the pathogenesis of AD is highlighted by the results of this article.

We noted that previous studies of microarray datasets applied DE genes with different thresholds of significance including a *q-*value (false discovery rate) with threshold of 0.2 (Rhodes et al., 2002), *p*-value with threshold of 0.01 (Bhargava et al., 2013) and so on. However, the different threshold value chosen for DE genes can reflect the different disease models (Dumur et al., 2004; O’dushlaine et al., 2009). Thus, a significant pathway using DE genes with a smaller threshold might reflect a role for less DE genes of larger effects and a significant pathway enriched by DE genes with a larger threshold may reflect the role of more DE genes with smaller effects (O’dushlaine et al., 2009; Cheng et al., 2018a). In this article, the DE genes with the cut-off of *p*-value 1.00*E*-04 were selected to perform the pathway analysis for exploring the relationship between immune system-mediated actions and Alzheimer’s disease.

## Materials and Methods

### Data

The microarray database, Gene Expression Omnibus database (GEO database)^1^ (Davis and Meltzer, 2007), was searched for datasets related to AD. Focusing our search on hippocampus brain of human as objects, we identified four publicly available microarray datasets of AD. The gene expression data and detail information about the collecting datasets were available through GEO accession numbers GSE1297 (Blalock et al., 2004), GSE5281 (Liang et al., 2007, 2008; Readhead et al., 2018), GSE28146 (Blalock et al., 2011), and GSE48350 (Berchtold et al., 2008, 2013; Astarita et al., 2010; Cribbs et al., 2012; Sarvari et al., 2012; Blair et al., 2013). Some details about the four microarray datasets of AD are given below.

Dataset GSE1297 was contributed by Blalock et al. (2004). The investigation explored gene expression data on hippocampal brain of nine controls, 22 AD cases and 31 subjects in total. The participants of this microarray dataset were aged from 65 to 110 years with median age 85 years. The platform of GSE1297 was Affymetrix Human Genome U133A Array (GPL96). For the aim of our investigation we used the data of all 31 samples.

Dataset GSE5281 was provided by Liang et al. (2006). It collected 161 individual brain specimens from three AD Centers including the Washington University Alzheimer’s Disease Centers, the Duke University Alzheimer’s Disease Centers and the Arizona Alzheimer’s Disease Centers. The participants of this microarray dataset were aged from 61 to 101 years with median age 79 years. The platform of GSE5281 was Affymetrix Human Genome U133A Array (GPL570). The study contained gene expression levels on six brain regions including hippocampus, posterior cingulate, entorhinal cortex, primary visual cortex, medial temporal gyrus, and superior frontal gyrus. For the aim of our study, we used only data on the brain regions of the hippocampus. The GSE5281 contained 23 samples on hippocampal brain, of which 10 samples were AD cases and 13 samples were controls.

Dataset GSE28146 was provided by Blalock et al. (2011) for the aim of exploring the association between AD and the distinction of gray and white signatures on hippocampus. It collected 30 individual brain specimens including eight controls and 22 AD cases. The participants of this microarray dataset were aged from 65 to 101 years with median age 86 years. The platform of GSE28146 was Affymetrix U133 Plus 2.0 array (GPL570). We use the dataset of 30 samples in total.

Dataset GSE48350 was provided by Berchtold et al. (2014). The datasets contained gene expression levels on four brain regions including entorhinal cortex, hippocampus, post-central gyrus, superior frontal cortex. The participants of this microarray dataset of AD were aged from 20 to 97 years with median age 69 years. The platform of GSE48350 is Affymetrix U133 Plus 2.0 array (GPL570). For the aim of our analysis, we used only data on the brain regions of the hippocampus. Detailed information about the four datasets is presented in Table 1.

**Table 1:** Patient and sample characteristics of Alzhemer’s gene expression datasets.

Characteristics	GSE1297	GSE5281	GSE28146	GSE48350
No. of participants	31	23	30	62
Group, AD:Control	22:09	10:13	22:08	19:43
Median age, ye	85 (65–110)	79 (62–101)	86 (65–101)	69 (20–97)
ars (range)				
Gender, M:F	13:18	16:07	12:18	32:30
Platforms	GPL96	GPL570	GPL570	GPL570


*GPL96, affymetrix human genome U133A array; GPL570, affymetrix human genome U133 Plus 2.0 array.*

We downloaded the series matrix files of the four different microarray expression profilings and the four datasets on AD were pre-processed by the authors. We applied log2 transform for the datasets. R packages including hgu133a.db (Carlson, 2016a) and hgu133plus2.db (Gautier et al., 2004; Carlson, 2016b) were used to annotate the datasets. The expression data of the probe sets corresponding to more than one gene were deleted. If a gene was mapped by more than one probe set, the average value of these probe sets was calculated. Then the function MetaDE.merge of MetaDE package (Gentleman et al., 2004; Wang et al., 2012) was used to extract the same genes from all studies. After that, the function MetaDE.filter of MetaDE package was used to filter out genes with very low gene expression level or small variation (cutoff = 0.10). Finally, the meta-analysis was conducted on 9519 target genes in 146 samples (73 AD cases and 73 controls) to explore the relationship between immune system and AD.

### Meta-Analysis

Adaptively weighted Fisher’s method was used to detect the DE genes with *p*<1.00*E*-04. Adaptively weighted Fisher’s method assigned different weight to each individual study:

$$U_{g}(\omega_{g})=-\sum_{i=1}^{k}\omega_{ig}\ln(p_{ig}),{\;\omega }_{i}=0\;or\;1$$

where *i* represents the *i*th genetic study(*i* = 1,2,… , *k*), *k* represents the number of microarray studies, *g* represents the gene *g*, the *p*-value in the *i*th genetic study of gene *g* is represented by*^p_ig_^* , and assuming *p_ig_* ∼ *unif*(0,1), ω*_ig_* represents the specific weight assigned to the *i*th microarray study of gene *g* Thus, $U_{g}(\omega_{g})\sim Gamma(\sum_{i=1}^{k}\omega_{ig},1).$ Assuming ω*_g_* = (ω_1*g*_,ω_2*g*_,… , ω_*kg*_) and *W* = [ω_*g*_|ω*_ig_*=0 *or* 1], AW method searches ^ω_*g*_^ through *W* to find the best weights that provide the minimum final *p*-value.

### Pathway-Based Test

A pathway analysis was conducted based on the DE genes with thresholds of 1.00E - 04 using a pathway analysis web tool, the Web-based Gene Set Analysis Toolkit provided by Zhang et al. (2005). WebGestalt performs enrichment analysis by incorporating available functional and biological data (Zhang et al., 2005). Here, the Kyoto Encyclopedia of Genes and Genomes (KEGG) pathway in WebGestalt was selected to perform pathway analysis.

## Results

The number of DE genes with *p*<1.00*E*-04 detected by AW method was 586 (Supplementary Table 1). The top 10 DE gene KEGG pathways are listed in Table 2.

**Table 2:** Significant pathways by pathway analysis of differentially expressed (DE) genes detected by AW method in KEGG.

PathwayID	Pathway name	C	O	E	R	*P*-Value	FDR
hsa04721	Synaptic vesicle cycle	63	19	2.43	7.81	1.01E-12	3.06E-10
hsa00190	Oxidative phosphorylation	133	24	5.13	4.67	1.69E-10	2.57E-08
hsa05016	Huntington’s disease	193	26	7.45	3.48	1.94E-08	1.96E-06
hsa03050	Proteasome	44	12	1.69	7.06	6.04E-08	4.57E-06
hsa05120	Epithelial cell signaling in *Helicobacter pylori* infection	68	14	2.62	5.33	2.22E-07	1.35E-05
hsa05012	Parkinson’s disease	142	19	5.48	3.46	1.90E-06	9.59E-05
hsa05010	Alzheimer’s disease	171	21	6.60	3.18	2.25E-06	9.72E-05
hsa05130	Pathogenic *Escherichia coli* infection	55	11	2.12	5.18	6.13E-06	0.000232
hsa05110	*Vibrio cholerae* infection	51	10	1.96	5.07	1.96E-05	0.000661
hsa04145	Phagosome	154	17	5.94	2.85	8.43E-05	0.002554


*The pathways are ordered by their *p*-values. C, represents the number of reference genes in the category; O, represents the number of genes in the gene set and also in the category; E, represents expected number in the category; R, represents the ratio of enrichment.*

The significant pathways were associated with some specific diseases including Huntington’s disease, AD and Parkinson’s disease. And these enriched pathways were connected mainly with the immune system, like Epithelial cell signaling in *Helicobacter pylori* infection pathway, *Vibrio cholerae* infection pathway, Pathogenic *Escherichia coli* infection pathway, and Phagosome pathway. The results of the meta-analysis suggested that AD is connected with the immune system.

## Discussion

The differentially expressed genes with threshold of *p*<1.00*E*-04 in the meta-analysis were reported. A pathway analysis in KEGG of the DE genes detected by AW method was conducted and the top 10 significant KEGG pathways were reported. Most of pathways were connected with neuro-diseases and the immune system. Here, we further compared our investigation based on the meta-analysis of microarray datasets with other studies.

Stopa et al. (2018) analyzed six healthy controls and seven patients of advanced AD using transcriptome-wide Affymetrix microarrays, and they reported immune system and metabolic pathways such as cytokine, interferons, cell adhesion, JAK-STAT, acute phase response, and mTOR pathway. However, Cribbs et al. reported that the extent of immune gene upregulation in AD was modest to the robust response apparent in the aged brain (Cribbs et al., 2012).

Using the DE genes with *p*<1.00*E*-04 of meta-analysis methods, we reported significantly enriched top 10 KEGG pathways and discovered that the pathways were associated with neuro-diseases like Huntington’s disease pathway (hsa05016), Parkinson’s disease pathway (hsa05012), and Synaptic vesicle cycle pathway (hsa04721). Furthermore, most DE genes identified by AW methods were enriched in the immune system including Epithelial cell signaling in *H. pylori* infection (hsa05120), Pathogenic *E. coli* infection (hsa05130), *V. cholerae* infection (hsa05110), and Phagosome (hsa04145).

The infection of *H. pylori* associated with AD was investigated using histology for diagnosis (Kountouras et al., 2006). The study showed that the pathophysiology of AD was influenced by *H. pylori* infection through one of the following mechanisms: (1) *H. pylori* may produce reactive oxygen metabolites and lipid peroxides which accelerate the occurrence of AD (Malaguarnera et al., 2004). (2) Increasing platelet-leukocyte aggregation and platelet reactivity (Kountouras et al., 2002). Platelets are a key component of amyloid which contributes to AD and causes the occurrence of senile plaque (Kountouras et al., 2006). (3) The cell apoptotic process might be influenced by *H. pylori* and the cell death has a close relationship with neurodegenerative diseases (neuro-diseases) such as AD (D’Andrea, 2005). (4) *H. pylori* may release a large amount of vasoactive substances and proinflammatory, such as eicosanoids, cytokines and acute phase proteins connected with a sea of disorders of the nervous system including AD (Kountouras et al., 2002). (5) *H. pylori* might contribute to down syndrome that drives the early onset of the neuro-diseases such as AD (Hallam et al., 2000). *E. coli* has been found to be closely associated with AD, and *E. coli* LT and LT (R192G) have been used as mucosal adjuvants to treat AD in mice (Lemere et al., 2002). Rheumatoid arthritis is closely related to AD and anti-inflammatory agents might be beneficial for AD (McGeer et al., 1996). In this article, we discovered that AD may be related to *H. pylori* infection, *E. coli* infection, Rheumatoid arthritis through pathway analysis of KEGG. Moreover, we also found that AD may be related to *V. cholerae* infection.

In summary, AW meta-analysis method was used to detect the DE genes with strict threshold of p < 1.00E - 04. The study reported the top ten significantly enriched pathways of the DE genes detected by AW method and our results show that these DE genes are significantly enriched in immune pathways.

## Availability of Materials and Data

All data used in this paper are fully publicly available without any restriction.

Dataset GSE1297 is available from the link of https://www.ncbi.nlm.nih.gov/geo/query/acc.cgi?acc=GSE1297.

Dataset GSE5281 can be got from the URL of https://www.ncbi.nlm.nih.gov/geo/query/acc.cgi?acc=GSE5281.

Dataset GSE28146 is available at https://www.ncbi.nlm.nih.gov/geo/query/acc.cgi?acc=GSE28146.

Dataset GSE48350 can be got from the URL: https://www.ncbi.nlm.nih.gov/geo/query/acc.cgi?acc=GSE48350.

## Author Contributions

SJ provided the guidance during the whole research. NW collected the data. LX carried out data analysis. YZ and LX wrote the manuscript.

## Conflict of Interest Statement

The authors declare that the research was conducted in the absence of any commercial or financial relationships that could be construed as a potential conflict of interest.

## Footnotes

1 https://www.ncbi.nlm.nih.gov/geo/
